# Working memory training improves episodic memory in older people: transfer based on controlled retrieval processes

**DOI:** 10.3389/fpsyg.2024.1314483

**Published:** 2024-03-20

**Authors:** Patricia Zamarreño, Pedro M. Mateos, Alberto Valentín

**Affiliations:** Faculty of Psychology, University of Salamanca, Salamanca, Spain

**Keywords:** older adults, cognitive training, episodic memory, working memory, recollection

## Abstract

**Introduction:**

The results of working memory (WM) training to improve episodic memory in older people are inconsistent. This inconsistency could be due to the fact that the episodic memory tests used do not share the same cognitive resources as the trained WM task. The aim of this study was to assess whether performance on an episodic memory test will improve only when this test requires controlled processes of retrieval of information from secondary memory or recollection, similar to the processes exercised during WM training.

**Method:**

Fifty-five people over 60 years of age participated in the study: 27 were randomly assigned to the experimental group (EG) and the rest to the control group (CG). The EG was trained in complex span tasks. Before and after training, both groups were tested on episodic memory tests (a verbal and a visuospatial recognition test) and WM span tasks (reading, digit and spatial location).

**Results:**

ANOVAs revealed a greater improvement of recollection estimates in the EG than in the CG for both verbal recognition (*p* = 0.023) and visuospatial recognition (*p* = 0.014).

**Discussion:**

Our results provide support for a cognitive mechanism whose shared presence favored transfer from training on a WM task to a test of episodic memory. Consistent with our predictions, training on complex span tasks improved performance on recognition tests only when recall required a controlled search process in secondary memory, or recollection. We therefore stress the importance of identifying other cognitive resources that are susceptible to transfer from a training task to other untrained tasks. A better understanding of the phenomenon of transfer is crucial for the design of increasingly effective intervention programs for older people.

## 1 Introduction

The ability to recall past events, also known as episodic memory, plays an important role in the functional and cognitive performance of older people. Of all memory types, episodic memory is considered to be the most sensitive to aging (Grady, 2012; Nyberg et al., 2012; Friedman, 2013). Changes in episodic memory affect various activities of daily living of older people, such as managing finances, handling medications, maintenance of personal independence and autonomy, etc. (Mendonça et al., 2022). Given the progressive aging of the world’s population (Bloom and Luca, 2016), numerous studies in recent years have focused on cognitive training aimed at improving episodic memory functioning in older adults.

One approach followed by some of these studies is to train cognitive processes that support episodic memory (Schmiedek et al., 2010), with working memory (WM) and processing speed being the most commonly trained processes. The rationale for this form of training relies on a mechanism called transfer, which refers to the fact that improvements in trained cognitive ability contribute to the improvement of untrained cognitive constructs.

To date, studies have found inconsistent results on the efficacy of WM training in improving cognition in general (see for reviews Teixeira-Santos et al., 2019; Saba and Blanchet, 2020; Lima-Silva et al., 2022) and episodic memory in older people in particular. Regarding episodic memory, some studies have reported improvements (Schmiedek et al., 2010; Richmond et al., 2011; Penner et al., 2012; Heinzel et al., 2014), other studies have not found such improvements (Dahlin et al., 2008b; Brehmer et al., 2012) and finally, others have obtained improvements in some episodic memory tests but not in others (Buschkuehl et al., 2008; McAvinue et al., 2013; Ballesteros et al., 2014; Toril et al., 2016).

Beyond methodological considerations that might partly explain the variability in results (see Shipstead et al., 2012; Melby-Lervåg and Hulme, 2013; Shawn Green et al., 2019; Ophey et al., 2020), the effectiveness of training might depend on the transfer process itself. That is, it has been suggested that for transfer to occur, training tasks and untrained tasks need to share the same cognitive and/or neural processes (Dahlin et al., 2008a; Jaeggi and Buschkuehl, 2014; Wu et al., 2021). Thus, the failure of some studies at obtaining transfer from training could be due to the fact that the performance of the episodic memory tests used does not require the same cognitive resources as the trained WM task.

Therefore, it is necessary to identify which cognitive resources underlying the execution of a WM task could be transferred to performance on an episodic memory test. Several theoretical models have specified the cognitive resources involved in the performance in WM tasks. Thus, the dual component model developed by Unsworth and Engle (2007) proposes the action of two cognitive processes: an active attentional control process to retain information in primary memory and a controlled search process to retrieve information shifted to secondary memory.

In particular, this model suggests that, in the performance of complex span tasks, the participant must maintain an active representation in primary memory of serially presented items (e.g., letters, digits, etc.), through a continuous focus of attention. At the same time, the participant must solve a processing task (e.g., performing simple arithmetic operations) that interferes with this attentional processing. This interference causes both difficulties in the attention required to maintain items in primary memory and the displacement of those items to secondary memory. Therefore, retrieval of the displaced items requires a controlled search process. The participant must retrieve, in a controlled manner, relevant items from secondary memory while inhibiting other distracting items. From this point of view, training in complex span tasks could improve both the focusing of attention on primary memory information and the controlled retrieval of information shifted to secondary memory. Improvement in either of these processes could potentially transfer to performance on a test of episodic memory. Thus, Unsworth and Engle’s (2007) model offers two possible mechanisms of WM that could be shared by episodic memory. Without undermining the importance of the attentional component, in the present study, we focused specifically on the potential transfer of retrieval processes from secondary memory. That is, we wanted to test whether the transfer would be detected only in cases where episodic memory test performance also required controlled retrieval processes.

To detect transfer to an episodic memory test of trained retrieval processes, it is necessary for episodic recall to be based on a *recollection* process, free of any element of *familiarity*. A recognition test might be particularly suitable for this purpose. This type of test makes it possible to determine, through a process-dissociation procedure (Jacoby, 1991; Yonelinas, 2002; Yonelinas and Jacoby, 2012), to what degree recollection and familiarity each contribute to recall.

In the process-dissociation procedure, participants must study two lists of items and subsequently recognize the previously presented items under two recall conditions (McBride, 2007). One of these conditions is called *inclusion*. In this condition, participants must recall all items belonging to either of the two lists and reject items that have not been presented. Here, the two processes that contribute to recall are mixed, since the retrieval of these items may be because they are actually remembered (recollection) or simply due to the fact that they sound familiar. The other condition is the so-called *exclusion* condition. In this condition, participants must recall only items belonging to one of the two lists and reject not only items that have not been presented previously, but also items belonging to the other list. Since both lists were presented in the study phase, all items have the same degree of familiarity for the participant. Therefore, recalling the items from the target list can only be based on a process of recollection, i.e., on a controlled search process for information that was encoded in a certain context or list (Rudebeck et al., 2012; Koenig et al., 2015).

A similar rationale to the process-dissociation paradigm is shared by the local/global recognition test (Oberauer et al., 2003). Here, plain items (simple pictures, words, etc.) are presented in individual frames. The task of the participant is to recognize the old items regardless of the frame in which they were presented in the study phase (global recognition), or to recognize whether the item appeared in the same frame (local recognition). In the latter condition, the task requires recollection processes, i.e., it demands a retrieval of the link between the item and the spatial context in which it was encoded. In contrast, the global condition requires recognizing old items independently of the spatial context in which they appeared. Therefore, processes of familiarity and not only recollection also contribute to this recognition. In short, local recognition is akin to a condition of exclusion, and global recognition to a condition of inclusion.

The exclusion/local condition of a recognition test thus becomes a suitable resource for assessing the influence of WM training on specific retrieval processes. We predicted that repeated practice of complex span tasks, in that it promotes controlled search processes in secondary memory, would improve performance in an exclusion/local condition, which requires recollection processes. However, the practice of these tasks would have no effect on performance in an inclusion/global condition in which the retrieval of items does not depend exclusively on recollection processes but also on the familiarity of those items.

Confirmation of this prediction would complement the studies that found a correlation between WM capacity and performance on episodic memory tests (Mogle et al., 2008; Unsworth et al., 2009, 2013; Unsworth, 2010). Thus, in one of these studies (Oberauer, 2005), participants performed a series of WM tasks and a set of recognition tests with exclusion/inclusion conditions. Statistical analyses revealed a relationship between WM capacity and recollection estimates but showed no relationship between WM and familiarity.

Therefore, the main aim of the present research was to test whether WM training in older people differentially affected exclusion/local and inclusion/global conditions in two recognition tests. To test this objective, participants were assigned to either an experimental group (EG) or an active control group (CG). The EG received WM training consisting of the repetitive performance of complex span tasks. The CG received non-adaptive training consisting of the repetitive performance of perceptual speed tasks. Before and after training, both groups performed two recognition tests, one verbal and the other visuospatial. Our prediction was that the EG would improve their performance from pre-test to post-test more than the CG in the exclusion/local condition of the two recognition tests. We did not expect these from pre-test to post-test differences between groups in the inclusion/global condition of both tests.

An implicit assumption, in this and other research, was that WM transfer to other tasks was due to WM capacity enhancement produced by training (Shipstead et al., 2012). To directly test this assumption, we further aimed to test whether EG, compared to CG, would improve in other, non-directly trained WM tasks. To this end, we selected three WM tests: a complex reading span task and two simple inverse span, digit, and spatial location tasks. We predicted that the EG would improve their performance from pre-test to post-test more than the CG on all three WM tests. In other words, beyond the far transfer of training to an untrained cognitive domain (episodic memory), we wanted to assess the near transfer to the trained domain (WM).

## 2 Materials and methods

### 2.1 Participants

The study involved 60 older people who voluntarily enrolled in a computer-assisted cognitive training program. All participants were people over 60 years of age and living independently. Participants were randomly assigned to an EG and a CG, so that the groups were matched as closely as possible in terms of age, gender, and educational level. The study was carried out in several senior centers administered by the City Council of Salamanca (Spain) and in several neighborhood associations. All participants in the training program gave written informed consent prior to the start of the study. This study was approved by the Bioethics Committee of the University of Salamanca.

All participants completed the training sessions, but five were excluded from the data analysis. One was excluded for suspected severe depression (score above 9 points on the Yesavage Geriatric Depression Scale; Sheikh and Yesavage, 1986), one for suspected cognitive impairment (score below 24 points on the Mini-Mental State Examination, MMSE; Folstein et al., 1983) and one for taking medication that affects cognition. In addition, two participants failed more than two of the ten training sessions and were also excluded from the analyses. Finally, out of the 55 study participants, 27 belonged to the EG and 28 to the CG.

These groups did not differ significantly (*p* > 0.05) in the following descriptive variables (see Materials and testing): age, mood, global cognition, and subjective memory complaints. There were only differences in years of schooling (*p* = 0.05), where the CG had on average more years of schooling than the EG (see Table 1, section a). There were also no gender differences between the groups [χ^2^(1) = 0.525; *p =* 0.469].

**Table 1:** Means (SD) in the pre-test of the experimental and control groups in: a) descriptive variables and b) transfer measures.

	EG *N* = 27	CG *N* = 28
**a) DESCRIPTIVE VARIABLES**		
Gender (male/female)	7/20	5/23
Age	71.85 (6.47)	72.29 (7.13)
Years of schooling	14.59^*^ (2.37)	16.11 (3.17)
Mood	2.52 (2.64)	2.57 (1.79)
Global cognition	27.96 (1.48)	27.96 (1.35)
Memory complaints	3.63 (1.96)	4.02 (1.70)
**b) TRANSFER MEASUREMENTS**		
Verbal recognition (exclusion condition)	0.65 (0.91)	0.48 (0.76)
Visuospatial recognition (local condition)	0.61 (0.58)	0.86 (0.79)
Reading span	0.44 (0.13)	0.50 (0.13)
Digit span	0.84 (0.10)	0.85 (0.06)
Spatial localization span	0.84 (0.07)	0.83 (0.06)

*p = 0.05.

### 2.2 Design and overall procedure

We used a repeated measures pre-test and post-test design. This was a double-blind randomized controlled trial in which participants were randomly assigned to the EG or the CG. The training took place over 5 weeks, throughout 10 sessions, with a frequency of two sessions per week of approximately 50 min each. The sessions were conducted in groups with no more than 14 participants, belonging to the EG and the CG. In each session, the number of tasks was seven, with an expected completion time of 6/7 min per task. The order of presentation of the training tasks was varied in each session to avoid performance on a task being affected by its order of appearance. In addition, we considered that it also served to maintain the motivation of the participants.

Approximately 1 week before the training began, we conducted the pre-test. It was carried out in two sessions; in the first one, we collected the demographic characteristics of the participants and, in the second one, we assessed WM and episodic memory. One week after the end of the training, we performed the WM and episodic memory post-test. In both the pre-test and the post-test, we followed the same order of presentation of the evaluation tests.

### 2.3 Training sessions

In the following section, we describe the tasks used for the training of the EG and the CG.

#### 2.3.1 Complex span tasks

For WM training, we developed seven complex span tasks of the type commonly used to assess WM capacity. To convert the assessment tasks into training tasks we followed the guidelines of Chein and Morrison (2010), adjusting the difficulty level of each task to the participant’s performance. In this way, we developed seven tasks (see Supplementary Figures S1, S2), four verbal tasks (counting span, digit span, operation span, and lexical span), and three visuospatial tasks (matrix span, alignment span, and rotation span). As in typical complex span tasks, these training task consisted of retention of individual serially presented items such as digits (digit span), consonant letters (counting, operation and lexical span), spatial position of dots (matrix and alignment span) and spatial location of arrows (rotation span). While attending to the presentation of these items, the participant had to perform an interfering task such as number comparison (digit span), circle counting (counting span), arithmetic calculation (operation span), lexical decisions (lexical span), symmetry judgements (matrix span), circle alignment (alignment span) and letter rotation (rotation span). To create these tasks, the E-Prime software (Version 2; Schneider et al., 2012) was used. All tasks had five trial blocks of three trials each. Throughout all sessions, the first block of trials of each task always started with a difficulty level of two recall items. An algorithm adjusted the difficulty level of the following blocks. At the end of each block, the algorithm first evaluated the serial recall. If this recall was correct on all three trials, the algorithm then checked the percentage of correct processing tasks. If this percentage was 80% or more, the difficulty level of the next block was increased by one item. If it was less than 80%, the difficulty level was maintained. However, if the serial recall was incorrect on all three trials, the difficulty level of the next block was automatically decreased by one item. Finally, if the serial recall was correct in only one or two trials, the same level of difficulty was maintained in the next block. At the end of the three-trial block, the participant received feedback on their performance in terms of the number of serial items recalled and the percentage of correctly solved processing tasks.

It is noteworthy that we did not use the sequence of events per trial followed by many researchers (e.g., Chein and Morrison, 2010; Richmond et al., 2011; Shipstead et al., 2012; Harrison et al., 2013) which consists of ending with one of the serial items. In our sequence, the last event of the trial was always the processing task. In this way, we ensured that the last serial item of the trial did not remain in primary memory and had to be retrieved from secondary memory instead.

In order to facilitate the training of the older people who participated in the study, some changes were introduced in the first sessions that simplified the performance of the tasks. On the one hand, we simplified the task presentation format in the first session, presenting serial items and processing tasks separately. On the other hand, we varied the time of item presentation across sessions. In the first two sessions, the items were presented without time limit, remaining on screen until the participant’s response. From the third session onwards, the presentation of the serial items was set at 1000 ms and the presentation of the processing task at 3000 ms. As an exception, in the matrix span task and the rotation span task, these time constraints were introduced in the fifth session. A feature common to all sessions was that the duration of the inter-trial interval of each task was controlled by the participant by pressing a mouse button.

#### 2.3.2 Perceptual speed tasks

For the CG, a pseudo-training in perceptual speed tasks was designed through the E-Prime software (Version 2; Schneider et al., 2012). These were fake perceptual speed tasks as the time constraints characteristic of the execution of this type of task were not imposed on the participant.

We used seven tasks, four of verbal type and three of visuospatial type (see Supplementary Figures S3, S4 for a graphical representation of the tasks). The difficulty level of these tasks, unlike the WM tasks, increased over the sessions equally for all participants. That is, it did not depend on the individual performance of each participant. Thus, this group performed tasks that predictably had no effect on WM and episodic memory.

To avoid that any cognitive improvement of the EG over the CG could be due to different expectations of improvement between the two groups, the CG intervention conditions were identical to those of the EG. That is, we designed the intervention to be perceived by participants as a potentially effective cognitive intervention.

### 2.4 Materials and testing

To ascertain the demographic characteristics of the participants, they filled in a questionnaire with their age, gender, years of schooling as well as illnesses and medication intake. We assessed the frequency of subjective memory complaints about forgetting names, the loss of commonly used objects, doubt about having taken an action, forgetting intentions, and learning difficulties. We used the Spanish adaptation of the Yesavage Geriatric Depression Scale (Martínez et al., 2002) to assess mood stability, and the Spanish version of the Mini-Mental State Examination (Lobo et al., 1999) to assess global cognitive level.

To assess episodic memory, we used two tests (verbal recognition test and visuospatial recognition test) and to assess WM, three tests (spatial span backward test, digit span backward test and reading span test). All these tests were presented in paper and pencil format to avoid that lack of practice in the use of the computer could influence the assessment.

#### 2.4.1 Verbal recognition test

The test began with a study phase of two-word lists: list A presented orally, and list B presented visually, in both cases at a rate of one word per second. Participants listened to the words from list A and read the words from list B written on individual cards. Participants had to memorize the words from both lists.

Each list A and B consisted of 18 words belonging to three semantic categories. One of the three categories was shared by both lists. Of the 18 words, 12 were target words (four per category) and six were filler words (two from a category in list A, two from a category in list B and two from the category shared by both lists). Three filler words were presented at the beginning of each list and three at the end to avoid primacy and recency effects. These filler words were the same in both lists.

To avoid test–retest learning effects, we used two similar versions of this test. The assignment of each version to the pre-test or post-test was counterbalanced across participants. In one version, the categories for list A were *spices and herbs* and *tools,* and for list B were *clothing* and *kitchen utensils*. The shared category was *fruits*. These five categories were taken from the California Verbal Learning Test (CVLT) (Delis et al., 1987) in its Spanish adaptation (Benedet and Alejandre, 1998). As for the other version, the categories in list A were *musical instruments* and *furniture*, and those in list B were *transports* and *parts of a building*. The shared category was *animals*. These five categories were taken from the second version of the California Verbal Learning Test (CVLT II) (Delis et al., 2000) in its Spanish adaptation (Nieto et al., 2014).

To select the words for each category, we used normative data for categories in Spanish (Marful et al., 2015). We proceeded as follows. As a first step, we eliminated from each of our categories: compound words; polysemic words in Spanish that could belong to several categories (e.g., *clavo* as a spice or as a tool); words referring to commercial brands; and non-specific words (e.g., *aliño* as a spice). We also discarded from each category the three words/examples with the highest frequency of production in the database. We then selected the words per category according to their lexical availability index, so that all categories had one word with an availability above 25 and the rest between 10 and 25. All filler words had a lexical availability index higher than 25. Finally, we made a single randomization, common to all participants, of the order of presentation of the words (see Supplementary Tables S1, S3 for a presentation of the words used in the study phase of both versions).

The test proceeded with a recognition phase with two conditions: an inclusion condition and an exclusion condition. All participants first performed the inclusion condition and then the exclusion condition. In each condition, 27 words were presented orally, of which six belonged to list A (two from each category), six to list B (two from each category) and 15 were new words. The lexical availability of each category had a similar average according to the indexes of Marful et al. (2015). As new words, we selected three unstudied words from each category, all with similar lexical availability values, to make the categories comparable with each other. The lexical availability value of all new words was less than 10. This criterion was followed to facilitate the recognition task. We avoided, as a criterion, the selection of unseen words of high/moderate lexical availability, which would foreseeably have encouraged the occurrence of false recognitions. We made a single randomization of the order of presentation of the words. This order of presentation was kept constant for all participants (see Supplementary Tables S2, S4 for a presentation of the words used in each of the recall phases of both versions).

In the inclusion condition, the participant had to say ‘yes’ if the word belonged to either of the two lists studied and ‘no’ if it was a new word. ‘Yes’ answers were considered hits (if they were words belonging to either of the two lists) or false alarms (if they did not belong to either of the two lists). In the exclusion condition, the participant had to say ‘yes’ if the word belonged to list A and ‘no’ if it belonged to list B or was a new word. ‘Yes’ answers were considered hits (if they were words belonging to list A), false intrusion alarms (if they were words belonging to list B) or false alarms (if they did not belong to either list).

#### 2.4.2 Visuospatial recognition test

The test began with a study phase in which participants were presented with a set of simple geometric images that were difficult to verbalize. To present the images, we used individual sheets of DIN A4 paper. Each image appeared in one of the four quadrants into which the sheet was divided. The images were presented at a rate of 4–5 s each. The participant had to memorize both the shape of the image and its position in the quadrants of the sheet. A single randomization of the order of presentation of the images was made, with the restriction that the same number of images appeared in each quadrant. This order of presentation was maintained for all participants.

As in the verbal recognition test, to avoid test–retest learning effects, we designed two similar versions of this test. The assignment of each version to the pre-test or post-test was counterbalanced across participants. In each version, we used 20 images, of which 16 were taken from the Visual Reproduction II subtest of the Wechsler Memory Scale (Wechsler, 2004) and four images were taken from the Visual Retention Test (Benton, 1981). We used the latter four as filler images, presenting two at the beginning and two at the end to avoid primacy and recency effects (see Supplementary Figures S5, S7 for a graphical representation of the stimuli used in the study phase of both versions).

After the study phase, the test continued with a recognition phase with two conditions: a global recognition condition (similar to the inclusion condition of the verbal recognition test) and a local recognition condition (similar to the exclusion condition of the verbal recognition test). In both conditions, we presented the images to be recognized on individual sheets of DIN A4 paper. In each condition, we made a single randomization of the order in which the images were presented. These orderings were held constant for all participants. All participants performed the global condition first and then the local condition.

In the global condition, we presented 12 images. Six were *old* images, seen in the study phase, and six were *new*. Each image was presented individually in the center of the empty sheet, that is, not divided into quadrants. In this global condition, the participant had to say ‘yes’ if they thought the image was old and ‘no’ if they thought it was new. ‘Yes’ answers were considered hits (if they were old images) or false alarms (if they were new images) (see Supplementary Figures S6, S8 for a graphical representation of the stimuli used in each of the recall phases of both versions).

The local condition differed from the global condition in that the images were presented in one of the quadrants of each sheet. In this condition, we presented 14 images, of which 10 were *old* images (six in the same quadrant as in the study phase and four in different quadrants) and 4 were *new* images. In this local condition, the participant had to say ‘yes’ if they believed that the image appeared in the same quadrant as in the study phase, and ‘no’ if they believed that the image appeared in a different quadrant or was a new image. ‘Yes’ answers were considered hits (old images/same quadrant), false alarms (new images) or false intrusion alarms (old images/different quadrant).

To familiarize participants with how to perform the visuospatial test described above, participants carried out a shortened version of the same test. We used a total of 10 images selected exclusively from the Visual Retention Test (Benton, 1981): six were used in the study phase, and the remaining four in the recognition phase as new images.

#### 2.4.3 Working memory

To measure WM, three tests were used: a complex reading span test (Elosúa et al., 1996) and two simple backward span tests taken from the Wechsler Memory Scale (Wechsler, 2004), digit span and spatial location.

To increase the variability of scores in these tests, we made several modifications to the traditional way of application and correction. Regarding their application, in the present research, we used all the levels of difficulty of each of these tests. Moreover, we presented these levels in a randomized manner. For each test, we designed two random orders of presentation of the levels of difficulty. The assignment of each order to the pre-test or post-test was counterbalanced among participants. Regarding its correction, we used the ‘partial-credit unit score’ (Conway et al., 2005; Friedman and Miyake, 2005) instead of absolute scores.

### 2.5 Data analysis

Statistical analyses were performed using IBM SPSS Statistics software (version 26). All were based on a signification level of *α* = 0.05.

To test for training-related gains from the first to the last session, we conducted *t* -tests. To analyze the transfer of WM training to the assessment tests, we conducted two-factor analyses of variance (ANOVAs) with group (experimental vs. control) as a between-subjects factor and session (pre-test vs. post-test) as a within-subjects factor.

To measure more precisely the degree of recall in recognition tests, we used two sensitivity parameters derived from signal detection theory (Macmillan and Creelman, 1991): *d’*(n) and *d’*(i). We calculated *d’* values from the z-scores of hits and false alarms according to the equation: *d’*(n) = Hits - False Alarms. We used the score *d’*(n) as a measure of recall in the inclusion/global condition. We calculated the intrusion *d’* values from the z-scores of hits and false intrusion alarms according to the equation: *d’*(i) = Hits - False intrusion alarms (Oberauer, 2005). We used the score *d’*(i) as a measure of recall in the exclusion/local condition.

Concerning the WM tests, we calculated WM amplitude using the partial scoring procedure proposed by Conway et al. (2005). Thus, we scored as a unit the total number of items to be recalled, and as a proportion of that unit, the number of items recalled (regardless of whether the recall order was correct). Once the test was completed, we averaged the scores obtained in each of the levels, obtaining a final score that could vary from 0 to 1.

## 3 Results

We present two types of results on the impact of WM training. Firstly, those concerning the gains obtained by EG participants in performance on trained WM tasks. Secondly, results regarding the transfer of training gains to untrained tasks in episodic memory (far transfer). Thirdly, results regarding the transfer of training gains to untrained task in working memory (near transfer).

To avoid biases in the transfer results, we first checked that there were no differences between groups before training in transfer measures. One-way ANOVAs revealed no significant differences between groups on each of the dependent variables (all *p* > 0.05; see Table 1, section b, for means and standard deviations).

### 3.1 Effects of working memory training

To assess the statistical signification of training-related gains, we compared the performance of the EG in the second training session with that of the last training session (session 10). Multiple comparisons *t*-tests showed significant gains in the performance across all seven training tasks [*t*(26) = 10.70, *p* < 0.001, *d =* 2.00], both in the verbal [*t*(26) = 9.56, *p* < 0.001, *d =* 1.79] and visuospatial tasks [*t*(26) = 7.29, *p* < 0.001, *d =* 1.36].

We also analyzed each of the span tasks individually. The *t* -tests revealed significant gains in the performance across all tasks: counting span task [*t*(26) = 8.54, *p* < 0.001, *d =* 1.60]; digit span [*t*(26) = 6.31, *p* < 0.001, *d =* 1.18]; lexical span [*t*(26) = 7.31, *p* < 0.001, *d =* 1.37]; operation span [*t*(26) = 4.48, *p* < 0.001, *d =* 0.84]; alignment span [*t*(26) = 10.46, *p* < 0.001, *d =* 1.95]; matrix span [*t*(26) = 2.80, *p =* 0.005, *d =* 0.52]; and rotation span [*t*(26) = 3.56, *p* < 0.001, *d =* 0.67]. We obtained a large effect size (Cohen’s *d* > 0.80) in all training tasks except matrix span and rotation span, which yielded medium values (Cohen’s *d* > 0.50). In all cases, we used the correction factor of Hedges and Olkin (1985) to avoid small sample bias. In addition, we performed a Bonferroni correction for multiple comparisons for each individual *t* at α = 0.05 /7 = 0.007. This value is higher than the individual *p-*values, which corroborates its signification.

### 3.2 Far transfer

We analyzed the far transfer of training to two tests of episodic memory: a verbal recognition test and a visuospatial recognition test.

#### 3.2.1 Verbal recognition

**Figure 1:**
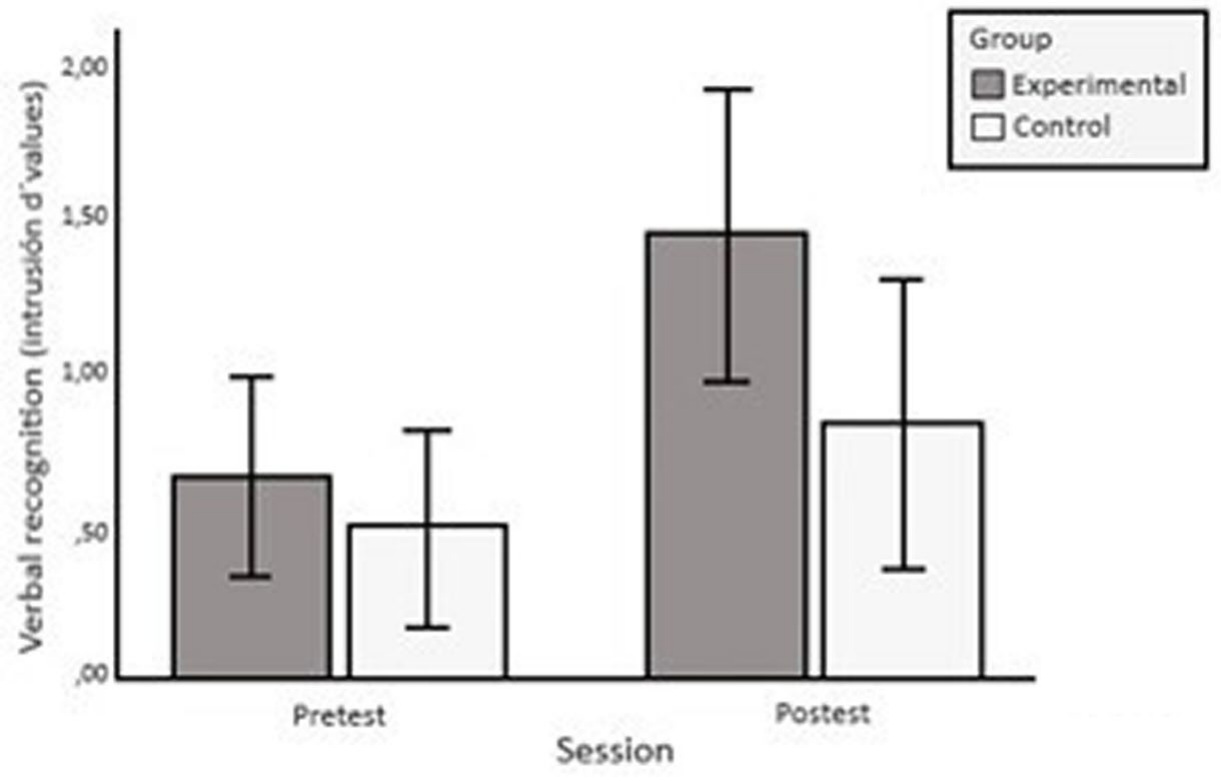
Pre-test and post-test means (intrusion *d’* values) of the EG and the CG in verbal recognition. The error bars represent a 95% confidence interval.

ANOVA on the *d’*(i) scores obtained in the exclusion condition revealed marginal effects of the group x session interaction, *F*(1, 53) = 3.908, *p* = 0.053, $\eta_{p}^{2}$ = 0.069. The trend of this interaction was in the direction of greater pre-test vs. post-test improvement in the EG than in the CG (see Figure 1). On the other hand, main effects were found for the session variable *F*(1, 53) = 25.028, *p* < 0.001, $\eta_{p}^{2}\;$ = 0.321 and not for the group variable, *F*(1, 53) = 2.213, *p =* 0.143, $\eta_{p}^{2}\;$ = 0.040.

It is possible that the marginal effects previously mentioned were due to other variables not initially included in the analysis. In this regard, participants’ previous school experience with verbal recall tasks is a particularly relevant variable. In this respect, the CG had more years of schooling than the EG (see Table 1). To eliminate the possible effect of this variable we conducted an ANCOVA introducing years of schooling as a covariate. The new analysis confirmed the group × session interaction we had predicted, *F*(1, 52) = 5.538, *p* = 0.022, $\eta_{p}^{2}\;$ = 0.096. That is to say, the interaction resulted in a greater pre-test vs. post-test improvement in the EG than in the CG.

ANOVA on the *d’*(n) scores obtained in the inclusion condition revealed no effects of the group x session interaction, *F*(1, 53) = 0.067, *p* = 0.797, $\eta_{p}^{2}\;$ = 0.001. That is, the EG did not show a greater pre-test vs. post-test improvement than the CG. On the other hand, it revealed no main effects for either the session variable, *F*(1, 53) = 2.598, *p =* 0.113, $\eta_{p}^{2}\;$ = 0.047, or the group variable, F(1, 53) = 0.043, *p =* 0.837, $\eta_{p}^{2}\;$ = 0.001.

#### 3.2.2 Visuospatial recognition

**Figure 2:**
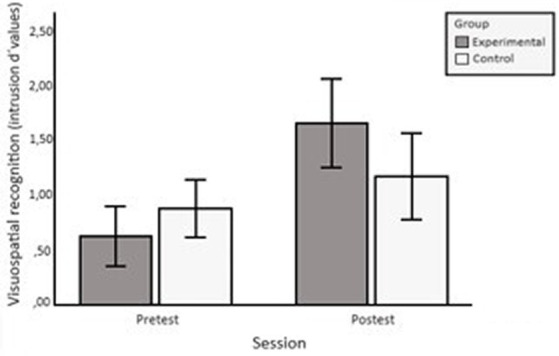
Pre-test and post-test means (intrusion *d’* values) of the EG and the CG in visuospatial recognition. The error bars represent a 95% confidence interval.

ANOVA on the *d’*(i) scores obtained in the local condition revealed effects of the group × session interaction, *F*(1, 53) = 6.395, *p* = 0.014, $\eta_{p}^{2}\;$ = 0.108. The interaction resulted in a greater pre-test vs. post-test improvement in the EG than in the CG (see Figure 2). Furthermore, main effects were found for the session variable *F*(1, 53) = 20.891*, p* < 0.001, $\eta_{p}^{2}\;$ = 0.283 and not for the group variable, *F*(1, 53) = 354, *p =* 0.555, $\eta_{p}^{2}\;$ = 0.007.

ANOVA on the *d’*(n) scores obtained in the inclusion condition revealed no effects of the group x session interaction, *F*(1, 53) = 1,093, *p* = 0.300, $\eta_{p}^{2}\;$ = 0.020. That is, the EG did not show a greater pre-test vs. post-test improvement than the CG. Moreover, it revealed no main effects for either the session variable, *F*(1, 53) = 0.022, *p =* 0.884, $\eta_{p}^{2}\;$ = 0.000, or the group variable, *F*(1, 53) = 1,168, *p =* 0.285, $\eta_{p}^{2}\;$ = 0.022.

### 3.3 Near transfer

We analyzed the near transfer of training to three WM tests: a complex reading span test and two simple backward span tests (digit and spatial location).

#### 3.3.1 Reading span

**Figure 3:**
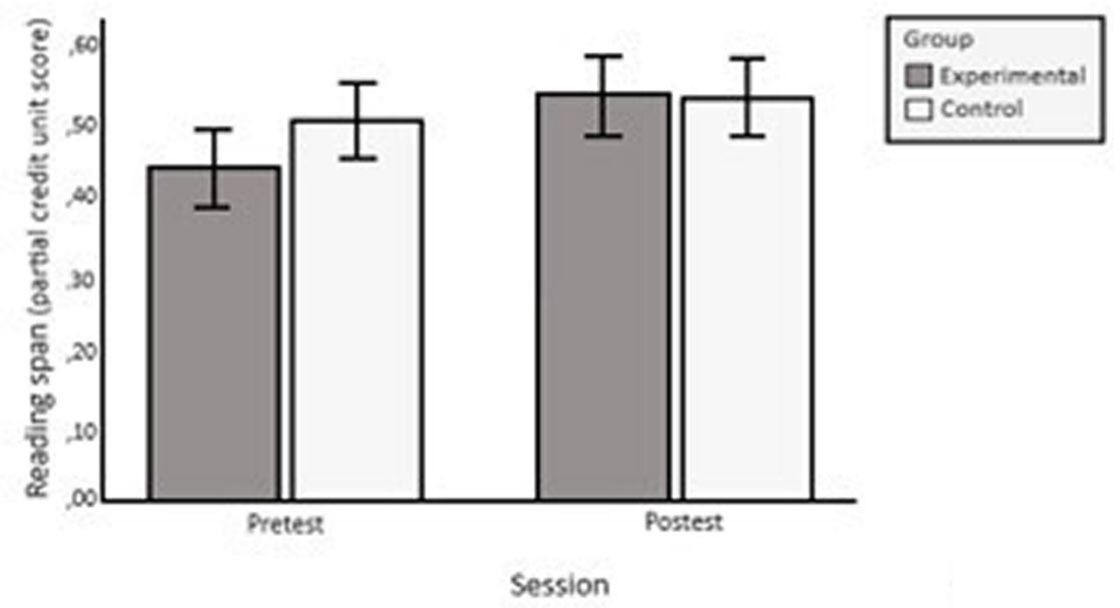
Mean values (partial-credit unit scores) pre-test and post-test of the EG and CG in Reading span. The error bars represent a 95% confidence interval.

ANOVA on span scores based on a ‘partial-credit unit scoring procedure’ revealed effects of the group × session interaction, *F*(1, 53) = 5.683, *p* = 0.021, $\eta_{p}^{2}\;$ = 0.097. The interaction resulted in greater pre-test vs. post-test improvement in the EG than in the CG (see Figure 3). In addition, main effects were found for the session variable *F*(1, 53) = 21.551, *p < 0*.001, $\eta_{p}^{2}\;$ = 0.289, and not for the group variable, *F*(1, 53) = 0.813, *p =* 0.371, $\eta_{p}^{2}\;$ = 0.015.

#### 3.3.2 Digit span

ANOVA on span scores based on a ‘partial-credit unit scoring procedure’ revealed no effects of the group × session interaction, *F*(1, 53) = 1.505, *p* = 0.225, $\eta_{p}^{2}\;$ = 0.028. That is, the EG did not show a greater pre-test vs. post-test improvement than the CG. In addition, it revealed no main effects for either the session variable, F(1, 53) = 0.411, *p* = 0.524, $\eta_{p}^{2}\;$ = 0.008, or the group variable, *F*(1, 53) = 0.122, *p* = 0.728, $\eta_{p}^{2}\;$ = 0.002.

#### 3.3.3 Spatial location span

ANOVA on span scores based on a ‘partial-credit unit scoring procedure’ revealed no effects of the group × session interaction *F*(1, 53) = 0.634, *p* = 0.429, $\eta_{p}^{2}\;$ = 0.012. That is, the EG did not show a greater pre-test vs. post-test improvement than the CG. In addition, it revealed no main effects for either the session variable, *F*(1, 53) = 055, *p = 0*.815, $\eta_{p}^{2}\;$ = 0.001, although it did reveal main effects of the group variable, *F*(1, 53) = 7.711, *p* = 0.008, $\eta_{p}^{2}\;$ = 0.127.

To estimate the effect size of the transfer between pre-test and post-test, we calculated Cohen’s *d* (Cohen, 1988) separately for each outcome variable in each group using individual *t*-tests. We used Hedges and Olkin’s (1985) correction factor to avoid small sample bias. We obtained large effect sizes for the EG in the local visuospatial recognition condition (*d* = 0.906), in the verbal recognition exclusion condition (*d* = 0.882) and in reading span (*d* = 0.860), and a small effect size in digit span (*d* = 0.145) and in spatial location (*d* = 0.237). We obtained a small effect size for the CG on these variables: local visuospatial recognition condition (*d* = 0.275), verbal recognition exclusion condition (*d* = 0.412), reading span (*d* = 0.319), digit span (*d* = −0.188). We obtained a medium effect size on spatial location (*d* = 0.522).

## 4 Discussion

In recent years, numerous studies have analyzed the efficacy of WM training to improve episodic memory in older people (see Hou et al., 2020 for a review). These studies yield inconsistent results in terms of far transfer, questioning the efficacy of this type of training.

It has been suggested that the efficacy of far transfer depends on training and transfer tasks sharing specific processes. In the present research, we have analyzed the contribution of a possible process shared by WM and episodic memory: controlled retrieval processes. To conduct this analysis, we created exclusion/local versus inclusion/global conditions in two recognition tests (one verbal and one visuospatial), with the assumption that only the first condition requires such controlled retrieval processes.

Regarding the verbal recognition test exclusion condition, the EG increased their scores from pre-test to post-test more than the CG. EG participants improved their efficiency in identifying which words belonged to a target List A, despite intrusions from List B words. To perform this test correctly, the participant must retrieve the previously encoded link between the items (words) and the context (list). That is, recall in the exclusion condition requires a process of conscious information retrieval, or recollection, that allows the participant to differentiate between target words and intrusions. This result, therefore, supports our hypothesis that training effects would be observed in an exclusion condition in which recall shares controlled retrieval processes with training tasks.

Similarly, in the local condition of the visuospatial recognition test, the EG increased their scores from pre-test to post-test more than the CG. The EG participants improved their ability to recognize whether a stimulus was a target stimulus (i.e., an image presented in the same location where it was encoded) or an intrusion (i.e., an image presented in a different location). To perform this test correctly, the participant had to retrieve the link between the image and the spatial context in which it was encoded. That is, recall in the local condition requires a process of conscious information retrieval or recollection. This result thus supports our hypothesis that training effects would be observed in a local condition in which recall shares controlled retrieval processes with training tasks.

On a purely empirical level, it should be noted that the pre-test vs. post-test differences between the EG and the CG were clearer in the visuospatial test than in the verbal test. In the latter, the improvement of the EG over the CG was only statistically marginal. This difference between the verbal and visuospatial tests may be due to the participant’s reading habits and school experience, which may have affected word recall more than recall of unusual images. The relevance of this parameter lies in the fact that, as we have already indicated, the years of schooling of the CG significantly exceeded those of the EG. In fact, once the effects of schooling were statistically controlled for, the improvement of the EG over the CG in the verbal recognition test was similar to that obtained in the visuospatial test. We can therefore conclude that, in both recognition tests, training is effective in the exclusion/local condition.

Regarding the inclusion/global condition, no pre-test vs. post-test differences were observed in the EG compared to the CG. That is, WM training did not improve the ability to correctly recall individual items (words/pictures) in a condition where the context (list/quadrant) in which those items were studied was irrelevant. In this type of condition, the participant could either recall an individual item with certainty by remembering the context in which it appeared or base the recall of that item on a sense of familiarity. In other words, both familiarity and recollection processes are involved in the inclusion/global condition, in an undifferentiated way. This result supports our hypothesis that training effects would not be observed in an inclusion/global condition in which recall does not rely exclusively on controlled retrieval processes.

Our results thus support a cognitive mechanism whose shared presence favors transfer from training on complex span tasks to recognition tests. More specifically, drawing on the theoretical framework of Unsworth and Engle (2007) and taking advantage of Jacoby’s (1991) process-dissociation paradigm, we have been able to verify the transfer of controlled secondary memory retrieval processes from trained WM tasks to the performance of episodic memory tasks.

In fact, this relationship between episodic and WM retrieval processes could be bidirectional. While conducting the present research, we learned of a study in which training to increase episodic memory retrieval efficiency improved performance on a running span task (Ma et al., 2020). The authors interpret its results in terms of the transfer of controlled retrieval processes from episodic memory to WM, per the aforementioned dual-component model.

Concerning testing whether WM training would improve WM capacity, that is, near transfer, the results were inconsistent. Regarding the reading span task, we found, as predicted, that the EG increased their scores from pre-test to post-test more than the CG. That is, EG participants improved their performance on an untrained WM test. This result supports our hypothesis that training would improve WM capacity.

However, in the simple backward, digit and spatial location span tests, our results were not as expected. In both tasks, the EG did not increase their scores from pre-test to post-test any more than the CG. That is, we could not detect changes in WM capacity using these tests. To what could this unexpected result be due? Are simple span tasks not suitable for measuring WM?

These questions are not easy to answer. The consideration of simple span tasks as a measure of WM capacity is a recurrent topic of discussion. Some researchers use simple span tasks as WM assessment test (Buschkuehl et al., 2008; Penner et al., 2012; McAvinue et al., 2013; Sandberg et al., 2016), while others use them as short-term memory assessment test (Borella et al., 2010, 2013, 2014; Chein and Morrison, 2010; Richmond et al., 2011; Heinzel et al., 2014). It should be noted, in this regard, that in the present research, we have used simple backward span tasks. These tasks involve a reordering of the presented items (Gignac et al., 2018) that simple direct tasks lack. That is, backward tasks require simultaneous storage and processing of information. This is the rationale for which we have considered them as WM tasks, a reasoning shared by other authors (see Oberauer et al., 2000), although there has been no shortage of those who question such logic (e.g., Engle et al., 1999).

In any case, the fact remains that our two simple span tasks were not affected by the training. Why did the training gains not transfer to these simple tasks? How do these tasks differ from complex span tasks? One obvious difference is that in complex tasks, but not in simpler ones, a processing task interferes with the storage task. It is assumed that this processing task consumes attentional resources causing deficiencies in the encoding of serial items to be remembered. Thus, in complex span tasks, the subsequent retrieval of such items from secondary memory becomes more demanding than retrieval in simple tasks. Perhaps, this is the reason why training focused precisely on controlled and effortful retrieval of information has influenced complex tasks and not these simple tasks.

Thus, the lack of transfer found in the simple span tasks can be interpreted in the same terms as the transfer found in the rest of the evaluation tasks. That is, transfer success depended neither on the similarity between the training and transfer tasks nor on the learning of useful strategies in the execution of both (see, nonetheless, Dunning and Holmes, 2014; Forsberg et al., 2020; Pergher et al., 2020 for the importance of strategy learning during the performance of WM training tasks). Rather, success depended on whether the performance of the training and transfer tasks both required the same specific processing process: the controlled retrieval of information from secondary memory.

Some methodological limitations should be taken into account when interpreting our results. The main limitation is that we used a relatively small sample size. Therefore, the results would need to be replicated with larger samples. Also, the sample was unbalanced in terms of the participants’ gender, with a higher proportion of women. This reflects the fact that women are more likely to volunteer for studies than men. This gender imbalance in cognitive training studies seems not to have changed in the last decade (see, for example, the reviews of Lampit et al., 2014; Yu et al., 2023). Furthermore, there is evidence of gender differences in brain aging (Armstrong et al., 2019). Therefore, the results would need to be replicated with more gender-balanced samples to be able to generalize our findings to both genders. On the other hand, in an attempt to facilitate the verbal recognition task for older people, we used words of low lexical availability as intrusions. This resulted in almost a ceiling effect in the verbal recognition task. Thus, in future research with older adults, words of higher lexical availability may be necessary to elicit higher rates of intrusions.

The efficacy of training on the complex span task, but not on the simple span tasks, requires further comment. It has been suggested from life-cycle psychology that cognitive training in healthy older people might affect so-called pragmatic abilities, such as verbal knowledge, as opposed to mechanical abilities, such as spatial orientation (Baltes et al., 1999). Thus, one possibility, not examined in the present study, is that the effectiveness of training only on the reading span test, and not on the digit span and spatial location tests, might be due in part to the verbal nature of the former. However, factor analyses of working memory abilities show that, although spatial working memory is different from both verbal and numerical working memory, there are no differences that justify the separation between the latter two (Oberauer et al., 2000). There is therefore a clear need for further research linking the effectiveness of cognitive training to the specific content of the working memory tests used in the assessment.

## 5 Conclusion

In conclusion, the present research provides evidence for the efficacy of cognitive training in older people. In contrast to studies questioning such efficacy (McCabe et al., 2016; Melby-Lervåg et al., 2016; Guye and Von Bastian, 2017; Sala et al., 2018), our results thereby offer an optimistic view on the possibilities of cognitive training in older people. At the same time, they underline the importance of identifying shared processes between training and transfer tasks. From this point of view, it is not a question of testing whether the memory of older people can be improved, but of discovering which specific processes are critical for good memory performance and how these can be trained. The importance of other possible shared processes for transfer to take place has also been highlighted. In this regard, some researchers have emphasized the transfer of updating and attentional processes linked to primary memory through training in continuous span tasks (e.g., Dahlin et al., 2008a,b) and n-back tasks (e.g., Jaeggi et al., 2008). We believe that research of this kind will prove crucial in expanding our understanding of the possibilities and limitations of cognitive training.

## Data availability statement

The original contributions presented in the study are included in the article/Supplementary material, further inquiries can be directed to the corresponding author.

## Ethics statement

The studies involving humans were approved by Comité de Bioética de la Universidad de Salamanca. The studies were conducted in accordance with the local legislation and institutional requirements. The participants provided their written informed consent to participate in this study.

## Author contributions

PZ: Writing – review & editing, Writing – original draft. PM: Writing – review & editing, Writing – original draft. AV: Writing – review & editing, Writing – original draft.
