# Supplementary material for: Working memory training improves episodic memory in older people: transfer based on controlled retrieval processes
Source: Front Psychol. 2024 Mar 20;15:1314483. doi: 10.3389/fpsyg.2024.1314483 (PMC10987720; doi:10.3389/fpsyg.2024.1314483)
Supplement: Supplementary file 1 [file Data_Sheet_1.docx]

# ***Supplementary figures and tables***

1. Training tasks:

- complex verbal span tasks (Figure 1)
- complex visuospatial span tasks (Figure 2)
- verbal perceptual speed tasks (Figure 3)
- visuospatial perceptual speed tasks (Figure 4)

1. Verbal recognition tests:

- study phase (Tables 1 - 3)
- recognition phase (Tables 2 – 4)

1. Visuospatial recognition tests:

- study phase (Figures 5 - 7)
- recognition phase (Figures 6 – 8)

## **FIGURE 1. Complex verbal span tasks used for working memory training.**

**Operation span task**

**
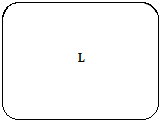
**
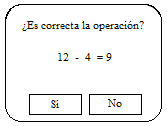

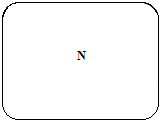
**
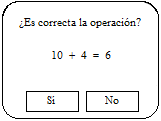
**
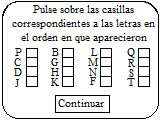


**Counting span task**


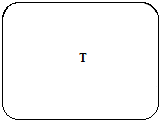
**
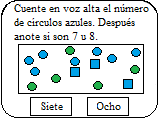

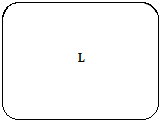

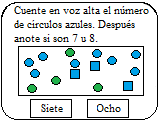
**
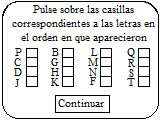


**Lexical span task**

**
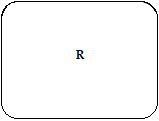

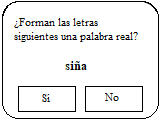

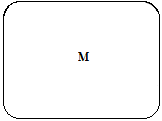

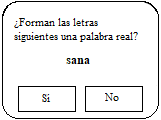
**
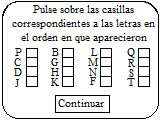


**Digit span task**


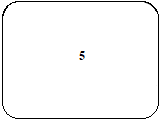

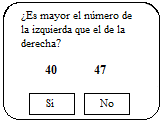

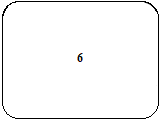
*
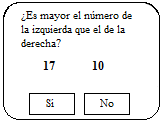

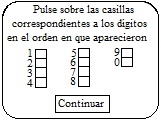
*

*Notes:* For all tasks, the lowest difficulty level (two-item serial recall) is shown. In the lexical span task, the words used are two-syllable, four-letter nouns with a frequency of use in Spanish of less than 2 per million (Pérez, Alameda and Cuetos, 2003).

## **FIGURE 2.** **Complex visuospatial span tasks used for working memory training.**

**Matrix span task**


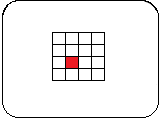

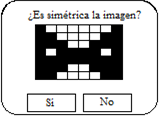

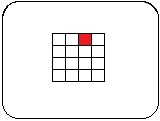

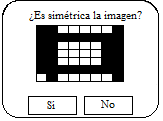

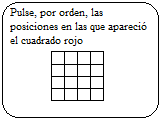


**Rotation span task**

**
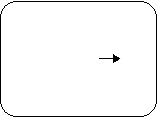

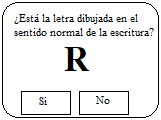

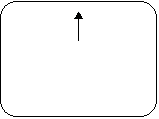

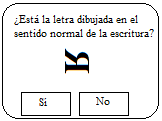

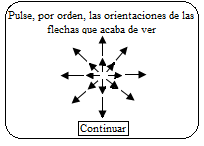
**

**Alignement span task**

**
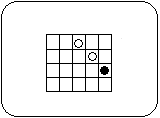

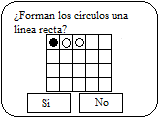

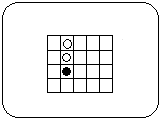
**
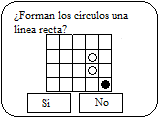
**
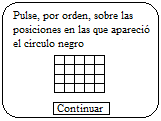
**

*Notes:* For all tasks, the lowest difficulty level (two-item serial recall) is shown. The stimuli used in the Matrix span task were taken from <http://englelab.gatech.edu/tasks.html> .

**FIGURE 3. Verbal perceptual speed tasks used for the training of the control group.**

**Inspection time task: letters**

**
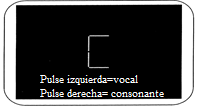

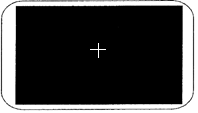

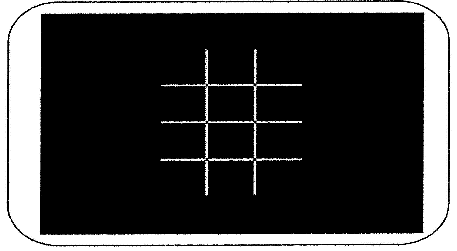

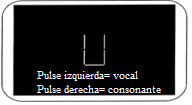

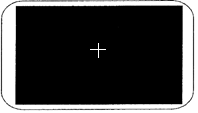
**

**Inspection time task: numbers**

**
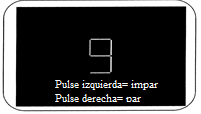

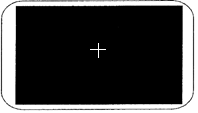

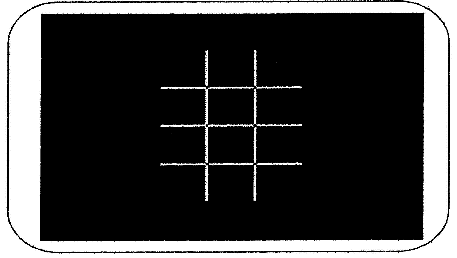

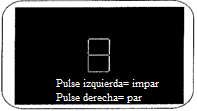

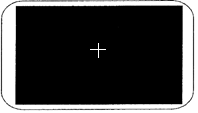
**

**Same/Different Judgment Task: Set of letters**

**
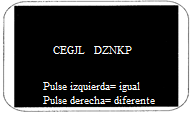

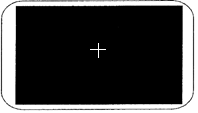

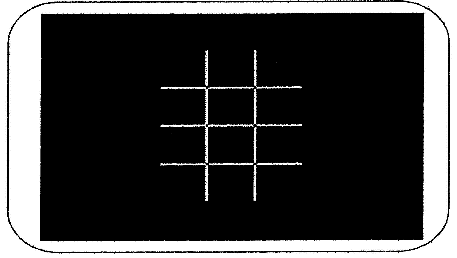

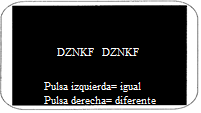

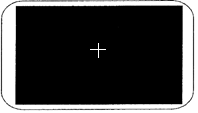
**

**Same/Different Judgment Task: Set of numbers.**

**
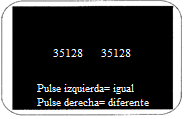

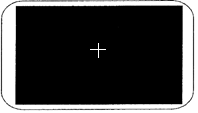

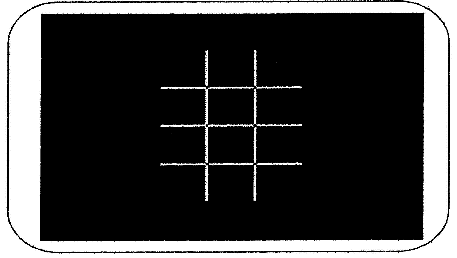

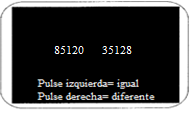

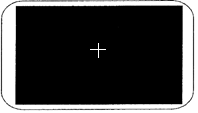
**

**FIGURE 4. Visuospatial perceptual speed tasks used for the training of the control group.**

**Same/Different Judgment Task: Images**

**
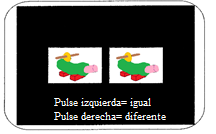

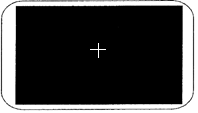

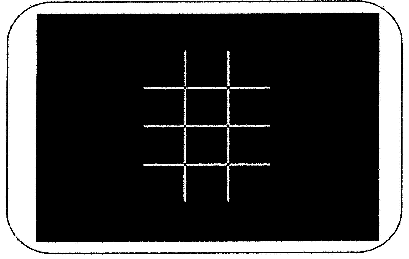

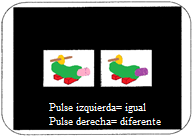

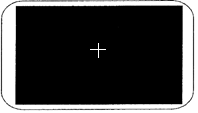
**

**Equal figures task.**

**
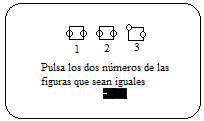

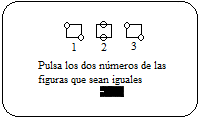
**

**Figures equal to the model task.**

**
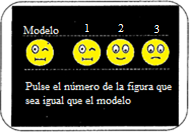

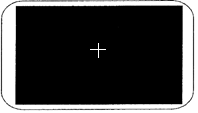

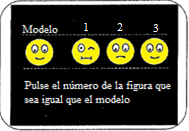

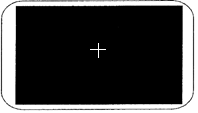

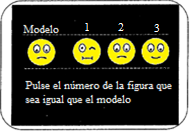
**

*Notes:* In ‘Same/Different Judgment task: Images’, the images were taken from Williams and Simons (2000). In the ‘Same Figures’ task, the figures were taken from Bonnardel (1970).

**TABLE 1. Words and categories used in the study phase of the recognition task (Version 1) grouped into their corresponding categories. The lexical availability of each word is also presented.**

| **LIST A** | | |  | **LIST B** | | |
| --- | --- | --- | --- | --- | --- | --- |
| **Categories** | **Studied words** | **Lex. Ava.** |  | **Categories** | **Studied words** | **Lex. Ava.** |
| Condimentos  (Spices and herbs) | 4. Azafrán (Saffron) | 27.15 |  | Prendas de  Vestir  (Clothing) | 4.Chaqueta (Jacket) | 28.35 |
|  | 9. Pimentón (Cayenne) | 20.28 |  |  | 9.Bufanda (Scarf) | 16.93 |
|  | 11.Canela (Cinnamon) | 16.99 |  |  | 11.Vestido (Dress) | 21.07 |
|  | 14.Azúcar (Sugar) | 13.91 |  |  | 14.Zapatos (Shoes) | 17.56 |
| **Average** | | **19.58** |  |  | **Average** | **20.98** |
| Herramientas  (Tools) | 5. Alicates (Nippers) | 34.22 |  | Utensilios de  Cocina  (Kitchen utensils) | 5.Cazo (Saucepan) | 26.72 |
|  | 7. Tenazas (Tongs) | 11.27 |  |  | 7.Plato (Dish) | 21.01 |
|  | 10.Taladro (Drill) | 21.33 |  |  | 10.Vaso (Glass) | 10.98 |
|  | 13.Tornillo (Screw) | 15.48 |  |  | 13.Olla (Pot) | 24.12 |
| **Average** | | **20.58** |  |  | **Average** | **20.71** |
| Frutas  (Fruits) | 6. Fresa (Strawberry) | 37.16 |  | Frutas  (Fruits) | 6.Mandarina (Tangerine) | 15.30 |
|  | 8. Albaricoque (Apricot) | 13.93 |  |  | 8.Cereza (Cheery) | 25.19 |
|  | 12.Limón (Lemon) | 14.51 |  |  | 12.Piña (Pineapple) | 13.06 |
|  | 15.Ciruela (Plum) | 14.49 |  |  | 15.Uvas (Grapes) | 15.44 |
| **Average** | | **20.02** |  |  | **Average** | **17.24** |
| Primacy words | 1.Orégano (Oregano) | 33.05 |  | Primacy words | 1.Orégano (Oregano) | 33.05 |
|  | 2.Jersey (Jersey) | 45.65 |  |  | 2.Jersey (Jersey) | 45.65 |
|  | 3.Sandía (Watermelon) | 31.64 |  |  | 3.Sandía (Watermelon) | 31.64 |
| **Average** | | **36.78** |  |  | **Average** | **34.93** |
| Recency words | 16.Ajo (Garlic) | 26.15 |  | Recency words | 16.Ajo (Garlic) | 26.15 |
|  | 17.Calcetines (Socks) | 36.10 |  |  | 17.Calcetines (Socks) | 36.10 |
|  | 18.Melón (Melon) | 37.00 |  |  | 18.Melón (Melon) | 37.00 |
| **Average** | | **33.08** |  | **Average** | | **33.08** |

*Notes:* Lex.Ava. = Lexical Availability. In each list, the number preceding each word indicates its order of presentation.

## **TABLE 2. Words and categories used in the inclusion and exclusion conditions of the recognition task (Version 1) grouped into their corresponding categories. The lexical availability of each word is also presented.**

| **INCLUSION CONDITION** | | | |  | **EXCLUSION CONDITION** | | | |
| --- | --- | --- | --- | --- | --- | --- | --- | --- |
| **Categories** | **Word types** | **Words** | **Lex.**  **Ava.** |  | **Categories** | **Word types** | **Words** | **Lex.**  **Ava.** |
| Condimentos (Spices and herbs) | Old | 1.Azafrán (Saffron) | 27.15 |  | Condimentos  (Spices and herbs) | Old | 8.Canela (Cinnamon) | 16.99 |
|  |  | 21.Azúcar (Sugar) | 13.91 |  |  |  | 11.Pimentón (Cayenne) | 20.28 |
|  | New | 4.Albahaca (Basil) | 5.90 |  |  | New | 15.Guindilla (Chilli) | 1.79 |
|  |  | 13.Romero (Rosemary) | 4.74 |  |  |  | 16.Comino (Cumin) | 6.58 |
|  |  | 14.Mahonesa (Mayonaisse) | 1.15 |  |  |  | 23.Mostaza (Mustard) | 2.54 |
| **Average** | | | **10.57** |  | **Average** | | | **9.64** |
| Herramientas  (Tools) | Old | 7.Taladro (Drill) | 21.33 |  | Herramientas  (Tools) | Old | 4.Alicates (Nippers) | 34.22 |
|  |  | 22.Tornillo (Screw) | 15.48 |  |  |  | 21.Tenazas (Tongs) | 11.27 |
|  | New | 10.Pala (Shovel) | 5.44 |  |  | New | 3.Lijadora (Sander) | 1.89 |
|  |  | 17.Serrucho (Handsaw) | 3.53 |  |  |  | 5.Tuerca (Nut) | 4.15 |
|  |  | 18.Radial (Angle grinder) | 1.82 |  |  |  | 24.Punta (Point) | 3.96 |
| **Average** | | | **9.52** |  | **Average** | | | **11.10** |
| Prendas de vestir  (Clothing) | Old | 6.Vestido (Dress) | 21.07 |  | Prendas de vestir  (Clothing) | New | 12.Sombrero (Hat) | 2.87 |
|  |  | 9.Zapatos (Shoes) | 17.56 |  |  |  | 14.Bañador (Swimsuit) | 2.71 |
|  | New | 3.Pañuelo (Neckerchief) | 2.71 |  |  |  | 26.Blusa (Blouse) | 9.13 |
|  |  | 5.Guantes (Gloves) | 9.27 |  |  | Intrusion | 19.Chaqueta (Jacket) | 28.35 |
|  |  | 19.Pijama (Pyjama) | 2.83 |  |  |  | 25.Bufanda (Scarf) | 16.93 |
| **Average** | | | **10.69** |  | **Average** | | | **12.00** |
| Utensilios de cocina  (Kitchen utensils) | Old | 11.Cazo (Saucepan) | 26.72 |  | Utensilios de cocina (Kitchen utensils) | New | 2.Cucharón (Ladle) | 7.24 |
|  |  | 27.Vaso (Glass) | 10.98 |  |  |  | 10.Horno (Oven) | 3.11 |
|  | New | 8.Colador (Strainer) | 3.77 |  |  |  | 17.Ensaladera (Salad bowl) | 2.22 |
|  |  | 15.Batidora (Beater) | 7.94 |  |  | Intrusion | 13.Plato (Dish) | 21.01 |
|  |  | 25.Jarra (Jug) | 1.63 |  |  |  | 18.Olla (Pot) | 24.12 |
| **Average** | | | **10.21** |  | **Average** | | | **11.54** |
| Frutas  (Fruits) | Old | 2.Uvas (Grapes) | 15.44 |  | Frutas  (Fruits) | Old | 7.Ciruela (Plum) | 16.99 |
|  |  | 16.Albaricoque (Apricot) | 15.93 |  |  |  | 20.Limón (Lemon) | 14.51 |
|  |  | 20.Fresa (Strawberry) | 37.16 |  |  | New | 1.Frambuesa (Raspberry) | 4.12 |
|  |  | 24.Mandarina (Tangerine) | 15.30 |  |  |  | 22.Banana (Banana) | 1.63 |
|  | New | 12.Níspero (Medlar) | 2.18 |  |  |  | 27.Nectarina (Nectarine) | 6.22 |
|  |  | 23.Higo (Fig) | 4.93 |  |  | Intrusion | 6.Cereza (Cherry) | 25.19 |
|  |  | 26.Aguacate (Avocado) | 5.59 |  |  |  | 9.Piña (Pineapple) | 13.09 |
| **Average** | | | **17.20** |  | **Average** | | | **11.68** |

*Notes:* Lex.Ava. = Lexical Availability. In each condition, the number preceding each word indicates the order of presentation.

**TABLE 3. Words and categories used in the study phase of the recognition task (Version 2) grouped into their corresponding categories. The lexical availability of each word is also presented.**

| **VERSION 2** | | | | | | |
| --- | --- | --- | --- | --- | --- | --- |
| **LISTA A (A LIST)** | | |  | **LISTA B (B LIST)** | | |
| **Categories** | **Studied words** | **Lex. Ava.** |  | **Categories** | **Studied words** | **Lex. Ava.** |
| Instrumentos musicales  (Musical instruments) | 4. Tambor (Drum) | 22.53 |  | Medios de transporte  (Transports) | 4.Avión (Plane) | 28.35 |
|  | 9. Clarinete (Clarinet) | 15.11 |  |  | 9.Furgoneta (Van) | 16.93 |
|  | 11.Acordeón (Accordion) | 17.15 |  |  | 11.Barco (Boat) | 21.07 |
|  | 14.Trompeta (Trumpet) | 29.17 |  |  | 14.Tractor (Tractor) | 17.56 |
| **Average** | | **19.58** |  |  | **Average** | **20.98** |
| Muebles de la casa  (Furniture) | 5. Sofá (Sofa) | 36.00 |  | Partes de un edificio  (Parts of a building) | 5.Escaleras (Stairs) | 26.72 |
|  | 7. Estantería (Shelving) | 23.79 |  |  | 7.Fachada (Facade) | 21.01 |
|  | 10.Cómoda (Chest of drawers) | 17.00 |  |  | 10.Sótano (Basement) | 10.98 |
|  | 13.Sillón (Armchair) | 23.27 |  |  | 13.Cimientos (Foundations) | 24.12 |
| **Average** | | **20.58** |  |  | **Average** | **20.71** |
| Animales  (Animals) | 6. Vaca (Cow) | 31.87 |  | Animales (Animals) | 6.Tigre (Tiger) | 15.30 |
|  | 8. Jirafa (Giraffe) | 14.96 |  |  | 8.Cerdo (Pig) | 25.19 |
|  | 12.Ratón (Mouse) | 13.77 |  |  | 12.Oveja (Sheep) | 13.06 |
|  | 15.Burro (Donkey) | 12.38 |  |  | 15.Conejo (Rabbit) | 15.44 |
| **Average** | | **20.02** |  |  | **Average** | **17.24** |
| Primacy words | 1.Violín (Violin) | 49.07 |  | Primacy words | 1.Violín (Violin) | 49.07 |
|  | 2.Autobús (Bus) | 29.63 |  |  | 2.Autobús (Bus) | 29.63 |
|  | 3.Elefante (Elephant) | 26.14 |  |  | 3.Elefante (Elephant) | 26.14 |
| **Average** | | **34.95** |  | **Average** | | **34.95** |
| Recency words | 16.Arpa (Harp) | 18.69 |  | Recency words | 16.Arpa (Harp) | 18.69 |
|  | 17.Bicicleta (Bicycle) | 28.21 |  |  | 17.Bicicleta (Bicycle) | 28.21 |
|  | 18.Oso (Bear) | 10.09 |  |  | 18.Oso (Bear) | 10.09 |
| **Average** | | **19.00** |  | **Average** | | **19.00** |

*Notes:* Lex.Ava. = Lexical Availability. In each list, the number preceding each word indicates its order of presentation.

## **TABLE 4. Words and categories used in the inclusion and exclusion conditions of the recognition task (Version 2) grouped into their corresponding categories. The lexical availability of each word is also presented.**

| **VERSION 2** | | | | | | | | |
| --- | --- | --- | --- | --- | --- | --- | --- | --- |
| **INCLUSIÓN** | | | |  | **EXCLUSIÓN** | | | |
| **Categories** | **Word types** | **Words** | **Lex. Ava** |  | **Categories** | **Word types** | **Words** | **Lex. Ava** |
| Instrumentos musicales (Musical instruments) | Old | 1.Trompeta (Trumpet) | 29.17 |  | Instrumentos musicales (Musical instruments) | Old | 8.Acordeón (Accordion) | 17.15 |
|  |  | 21.Clarinete (Clarinet) | 15.11 |  |  |  | 11.Tambor (Drum) | 22.53 |
|  | New | 4.Tuba (Tuba) | 2.98 |  |  | New | 15.Gaita (Bagpipes) | 2.61 |
|  |  | 13.Flautín (Piccolo) | 1.97 |  |  |  | 16.Corneta (Cornet) | 1.57 |
|  |  | 14.Bandurria (Bandurria) | 7.22 |  |  |  | 23.Armónica (Harmonica) | 2.54 |
| **Average** | | | **11.29** |  | **Average** | | | **9.28** |
| Muebles de la casa  (Furniture) | Old | 7.Estantería (Shelving) | 23.79 |  | Muebles de la casa  (Furniture) | Old | 4.Sofá (Sofa) | 36.00 |
|  |  | 22.Sillón (Armchair) | 23.27 |  |  |  | 21.Cómoda (Chest of drawers) | 17.00 |
|  | New | 10.Taburete (Stool) | 5.18 |  |  | New | 3.Taquillón (Locker) | 1.66 |
|  |  | 17.Ropero (Wardrobe) | 2.83 |  |  |  | 5.Aparador (Cupboard) | 5.65 |
|  |  | 18.Alacena (Cupboard) | 1.22 |  |  |  | 24.Perchero (Coat stand) | 2.42 |
| **Average** | | | **1.26** |  | **Average** | | | **12.55** |
| Medios de transporte  (Transports) | Old | 6.Furgoneta (Van) | 20.01 |  | Medios de transporte  (Transports) | Intrusion | 19.Avión (Plane) | 26.86 |
|  |  | 9.Barco (Boat) | 15.45 |  |  |  | 25.Tractor (Tractor) | 11.53 |
|  | New | 3.Automóvil (Car) | 3.32 |  |  | New | 12.Camioneta (Pick up truck) | 3.09 |
|  |  | 5.Triciclo (Tricycle) | 6.85 |  |  |  | 14.Tranvía (Tram) | 6.46 |
|  |  | 19.Taxi (Taxi) | 2.27 |  |  |  | 26.Caravana (Caravan) | 3.89 |
| **Average** | | | **9.58** |  | **Average** | | | **10.37** |
| Partes de un edificio  (Parts of a building) | Old | 11.Escalera (Stairs) | 27.60 |  | Partes de un edificio  (Parts of a building) | Intrusion | 13.Fachada (Facade) | 15.46 |
|  |  | 27.Sótano (Basement) | 14.38 |  |  |  | 18.Cimientos (Foundations) | 18.52 |
|  | New | 8.Trastero (Storage room) | 2.26 |  |  | New | 2.Terraza (Terrace) | 7.25 |
|  |  | 15.Garaje (Garage) | 7.28 |  |  |  | 10.Tuberías (Piping) | 1.50 |
|  |  | 25.Patio (Patrio) | 1.41 |  |  |  | 17.Cornisa (Cornice) | 1.85 |
| **Average** | | | **10.59** |  | **Average** | | | **10.68** |
| Animales  (Animals) | Old | 2.Oveja (Sheep) | 13.51 |  | Animales  (Animals) | Old | 7.Ratón (Mouse) | 13.77 |
|  |  | 16.Burro (Donkey) | 12.38 |  |  |  | 20.Jirafa (Giraffe) | 14.96 |
|  |  | 20.Vaca (Cow) | 31.87 |  |  | New | 1.Gacela (Gazelle) | 2.84 |
|  |  | 24.Cerdo (Pig) | 17.36 |  |  |  | 22.Tortuga (Tortoise) | 3.24 |
|  | New | 12.Yegua (Mare) | 3.51 |  |  |  | 27.Jabalí (Wild boar) | 6.71 |
|  |  | 23.Mono (Monkey) | 2.63 |  |  | Intrusion | 6.Tigre (Tiger) | 29.48 |
|  |  | 26.Lobo (Wolf) | 6.16 |  |  |  | 9.Conejo (Conejo) | 10.43 |
| **Average** | | | **12.49** |  | **Average** | | | **11.63** |

*Notes:* Lex.Ava. = Lexical Availability. In each condition, the number preceding each word indicates the order of presentation.

**FIGURE 5. Images used in the study phase of the recognition task (Version 1).**


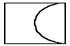


**1**


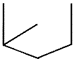


**2**


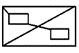


**3**


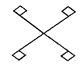


**5**


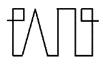


**6**


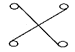


**7**


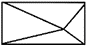


**9**


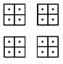


**17**


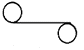


**13**


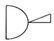


**10**

**14**


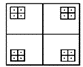


**11**


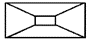


**15**


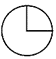


**19**


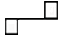


**4**


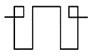

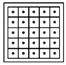


**18**

**20**

**12**


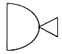


**16**

**82**


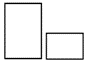

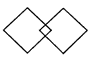

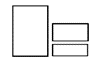


*Notes:* The number represents the order of presentation of each image. Images 1 and 2 are primacy stimuli and images 19 and 20 are recency stimuli.

**FIGURE 6. Images used in the global and local conditions of the recognition phase (Version 1).**

**GLOBAL CONDITION LOCAL CONDITION**

**Old New Old/same frame New Old/different frame**


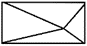

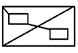

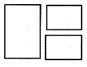


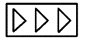

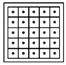
**2 1 1 4 2**


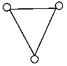

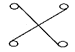

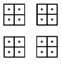

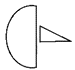


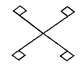
**3 4 3 8 5**


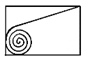

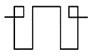

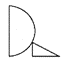


**7**  **5 6 12 10**

**8 6 7 14 13**

**10 9 9**

**11 12 11**

*Notes:* In each condition, the number preceding each image indicates the order of presentation

**FIGURE 7. Images used in the study phase of the recognition task (Version 2).**

**1**

**2**

**10**

**11**

**13**

**14**

**15**

**17**

**4**

**5**

**6**

**7**

**9**

**3**

**8**

**12**

**16**

**20**

**19**

**18**

**19**

*Notes:* The number represents the order of presentation of each image/quadrant. Images 1 and 2 are primacy stimuli and images 19 and 20 are recency stimuli.

**FIGURE 8. Images used in the global and local conditions of the recognition phase (Version 2).**

**GLOBAL CONDITION LOCAL CONDITION**

**Old New Old/same frame New Old/different frame**

**2 1 1 4 2**

**3 4 3 8 5**

**7 5 6 12 10**

**8 6 7 14 13**

**10 9 9**

**11 12 11**

*Notes:* In each condition, the number preceding each image indicates the order of presentation.

**SUPPLEMENTARY MATERIAL REFERENCES**

Bonnardel, R. (1970). BG3: Test de las figuras iguales [BG3: Equal figure Test]. Madrid: Mepsa.

Pérez, M. Á., Alameda, J. R., y Cuetos, F. (2003). Frecuencia, longitud y vecindad ortográfica de las palabras de 3 a 16 letras del Diccionario de la Lengua Española (RAE,1992) [Frequency, length and orthographic neighborhood of words from 3 to 16 letters from Diccionario de la Lengua Española (RAE, 1992)]. *Revista Electrónica de Metodología Aplicada*. 8(2), 1–10).

Williams, P. y Simons, D.J. (2000). Detecting Changes in Novel, Complex Three-dimensional Objects. *Vis cogn.* 7:1-3, 297-322. https://doi.org/10.1080/135062800394829
